# Editorial: Neurorehabilitative and regenerative methods involved in treating traumatic brain and spinal cord injuries, volume II

**DOI:** 10.3389/fnhum.2026.1911179

**Published:** 2026-07-17

**Authors:** Carolina Fiorin Anhoque, Walter Gomes da Silva Filho, Guilherme Peixoto Tinoco Arêas, Jader Vinícius da Silva Rocha, Fernando Zanela da Silva Arêas

**Affiliations:** 1 Laboratory of Neurorehabilitation and Neuromodulation, Universidade Federal do Espirito Santo, Vitoria, Brazil; 2 Postgraduate Program in Human Movement Sciences, Physiological Science Department, Universidade Federal do Amazonas, Manaus, Brazil; 3 Baylor Scott and White Research Institute (BSWRI), Dallas, TX, United States

**Keywords:** neurorehabiliation, neurotrauma, rehabilitation, spinal cord injury, traumatic brain injury

Traumatic injuries to the brain and spinal cord remain among the most devastating conditions worldwide, frequently resulting in long-term disability, loss of independence, and multifaceted rehabilitation needs (Gonçalves et al., 2025; Areas et al., 2019). Despite remarkable advances in acute medical care, the challenges associated with functional recovery underscore the urgency of developing innovative strategies capable of promoting neuroplasticity, neuroprotection, and tissue repair (Cramer, 2018; Arêas and Gonçalves, 2022).

In recent years, the field has witnessed rapid progress driven by emerging neuromodulatory approaches, biological therapies, and integrative rehabilitation protocols designed to restore neural function and improve quality of life (Hernandez-Navarro et al., 2025). Leveraging this momentum, this Research Topic, “*Neurorehabilitative and regenerative methods involved in treating traumatic brain and spinal cord injuries, volume II*,” highlights contemporary breakthroughs that bridge mechanistic insights with clinical applications in neurorehabilitation and regenerative medicine. It was conceived to showcase high-quality scientific contributions addressing the multifaceted nature of traumatic injuries to the central nervous system.

Extending the scope established in Volume I (Arêas et al., 2024), this second Research Topic highlights interdisciplinary perspectives, bridging basic science, technological innovation, and applied clinical research. The Research Topic brings together contributions examining cellular and molecular repair processes, neuromodulation-based interventions, sensorimotor rehabilitation paradigms, and the development of novel biomarkers and assessment tools. Collectively, these findings reflect the dynamic evolution of the field and the shared commitment to translating scientific discoveries into meaningful therapeutic advancements.

The articles compiled in this volume provide critical insights into mechanisms underlying functional recovery while simultaneously illustrating the diversity of methodologies currently shaping neurorehabilitation science. From the modulation of neuronal excitability and enhancement of neuroplasticity to bioengineered solutions aimed at structural regeneration, the research presented here underscores the expanding repertoire of strategies being tested to mitigate the sequelae of traumatic injuries. Particular emphasis is placed on integrating regenerative principles with rehabilitative practice, a paradigm increasingly recognized as essential to optimizing outcomes.

This convergence of approaches highlights that successful recovery relies not only on biological repair but also on targeted behavioral interventions that harness the nervous system's capacity for reorganization.

By assembling 13 contributions, including four original research articles (Liu, Zhang et al.; Shlifer et al.; Keleman et al.; Algaba-Vidoy et al.), three cases reports (Vieira de Melo et al.; Liu, Li et al.; Cordeiro et al.), three systematic reviews (Bhatti et al.; Wang et al.; Zhang et al.), one perspective (Moumeni), one review (Benavides et al.), and one study protocol (Hu et al.), that span preclinical experimentation, translational research, and clinical application, this Research Topic offers a timely update on emerging therapeutic possibilities.

The collective findings reinforce a central message: Progress in this field depends on cross-disciplinary collaboration and continuous methodological refinement. As our understanding of neurobiological recovery deepens, so does the potential to design more effective, personalized interventions. The studies published in this volume, therefore, not only advance current knowledge but also help define the next generation of rehabilitative and regenerative strategies for traumatic brain and spinal cord injuries.

Addressing factors associated with injury severity and neurological outcomes, Liu, Zhang et al. aimed to investigate risk factors by comparing patients with and without neurological impairment following thoracolumbar burst fractures in a cohort of 322 patients. Clinical, neurological (ASIA), and imaging data were analyzed, comparing patients with and without neurological deficits. Laminar fractures showed a stronger association with neurological impairment. Multivariate analysis identified laminar fractures, motor vehicle accidents, and falls as independent risk factors for neurological deficits. Furthermore, laminar fractures and falls demonstrated high predictive value for neurological involvement. These findings may support surgical decision-making and help optimize rehabilitation strategies for patients with thoracolumbar burst fractures.

Shifting from prognostic factors to therapeutic interventions targeting chronic sequelae of brain injury, Shlifer et al. conducted a retrospective study evaluating the effects of hyperbaric oxygen therapy (HBOT) on chronic neurocognitive symptoms in adults with post-concussion syndrome following pediatric traumatic brain injury. In total, 26 adults received at least 40 HBOT sessions, each consisting of 90 min of 100% oxygen exposure. Significant improvements were observed in global cognition, memory, executive function, attention, and information processing speed, while motor skills remained unchanged. Cognitive gains were independent of injury severity and time since injury, which averaged 23.6 years. Participants with mild and severe injuries showed similar responses, suggesting HBOT may facilitate long-term neurocognitive recovery after pediatric brain injury.

Complementing these findings on post-injury recovery, and emphasizing the importance of rehabilitation timing and intensity, Keleman et al. investigated the effects of extended early rehabilitation in patients with moderate and severe traumatic brain injury. In a prospective study involving 124 participants, the authors compared an intensified kinesitherapy protocol with standard rehabilitation. The findings demonstrated reductions in clinical complications, including the need for nasogastric tubes, tracheostomies, pressure ulcer management, and other supportive devices at discharge. Although mortality rates were similar between groups, the extended rehabilitation program proved safe and was associated with more favorable functional outcomes. These results reinforce the importance of early and intensive rehabilitation strategies following traumatic brain injury.

While the previous studies focused primarily on traumatic brain injury, the following contributions expand the discussion to spinal cord injury rehabilitation and emerging technological approaches. Algaba-Vidoy et al. explored the feasibility of combining transcutaneous spinal cord stimulation with robotic exoskeleton-assisted gait training in individuals with incomplete spinal cord injury. Using a case series design involving three participants, the authors assessed immediate neuromuscular and functional responses. This combined intervention proved to be technically feasible and safe, although responses varied substantially among participants. Some individuals demonstrated increased muscle activation and functional gains, while others exhibited more modest effects. The study highlights the potential of hybrid neurorehabilitation approaches and underscores the need for larger studies to identify the patients most likely to benefit from this strategy.

Providing a practical example of advanced technology-assisted rehabilitation, Vieira de Melo et al. investigated an 18-year-old male patient with traumatic incomplete paraplegia (L2, AIS C) who underwent 20 sessions of robotic exoskeleton training combined with conventional physiotherapy over 6 months. Neurological, functional, muscular, and bone health outcomes were assessed throughout the intervention. The results demonstrated neurological recovery, improved gait performance, and the ability to walk 300 meters using crutches. Hip extensor strength increased by 21.7%, and bone mineral density improved markedly, particularly in the lumbar spine. Functional improvements were associated with muscle strength recovery. Despite these benefits, persistent neuromuscular asymmetries and a substantial increase in visceral fat were observed, highlighting the necessity for comprehensive, multifaceted rehabilitation strategies.

Returning to the management of severe traumatic brain injury, Liu, Li et al. reported the case of a 39-year-old man with polytrauma and ultra-severe traumatic brain injury after a fall from height. The patient presented with a Glasgow Coma Scale score of 4, cerebral contusion, subarachnoid hemorrhage, skull-base fracture, traumatic wet lung, and trauma-associated coagulopathy. The authors applied the GHOST-CAP eight-dimensional goal-directed strategy, together with multimodal neurological monitoring, including cerebral blood flow, electroencephalography, cerebral oxygenation, and intracranial pressure-related assessment. The patient progressively improved, suggesting that this structured, physiology-guided approach may help manage complex interactions between brain protection and systemic critical care in severe trauma.

In parallel with advances in critical care management, neuromodulation techniques continue to emerge as promising tools for enhancing recovery following brain injury. Cordeiro et al. investigated the effects of transcranial direct current stimulation (tDCS) on functional and cognitive recovery in five men with severe traumatic brain injury during the subacute phase. Participants underwent five consecutive tDCS sessions targeting the left dorsolateral prefrontal cortex (DLPFC). Outcomes were assessed before and after treatment using cognitive, functional, pain, and mental health measures, including the MMSE, HADS-A, VAS, FIM, RLAS, and GOS-E. The results demonstrated substantial improvements across all assessed domains following the tDCS intervention, suggesting that tDCS may facilitate TBI rehabilitation and supporting its broader clinical application.

Beyond individual clinical studies, several review articles included in this Research Topic provide broader perspectives on factors influencing recovery and the effectiveness of emerging interventions. Bhatti et al. present a systematic review currently undergoing final formatting prior to publication, examining the impact of aging on locomotor recovery following traumatic spinal cord injury in animal models. A total of nine studies involving more than 340 animals were included. Across studies, older animals consistently demonstrated poorer locomotor recovery compared to their younger counterparts. The review highlights age as an important biological variable in spinal cord injury research and suggests that interventions such as exercise may help mitigate age-related impairments. The authors emphasize the need for future research investigating the mechanisms underlying reduced recovery and potential targeted therapeutic approaches.

Expanding the discussion of rehabilitation technologies across neurological populations, Wang et al. assessed 42 randomized controlled trials involving 1,678 stroke patients, comparing robot-assisted therapy with conventional upper limb rehabilitation. The results demonstrated significant improvements in motor function, grip strength, activities of daily living, and social participation with robotic therapy. The greatest benefits were observed in patients in the subacute phase of stroke and in those with severe impairments. Improvements in upper limb motor function and daily living activities exceeded minimal clinically important difference thresholds, indicating clinically meaningful recovery. Although gains in grip strength and social participation were statistically significant, their clinical relevance remains uncertain. These findings support the use of robotic rehabilitation in selected stroke populations.

In addition to robotic-assisted interventions, complementary therapeutic approaches are also being explored to address secondary complications after spinal cord injury. Zhang et al. conducted a systematic review evaluating the efficacy and safety of electroacupuncture (EA) for urinary incontinence following spinal cord injury. In total, 15 randomized controlled trials involving 1,394 patients were included. Compared to conventional rehabilitation alone, EA significantly reduced incontinence frequency and residual urine volume while improving urine output, voiding volume, bladder capacity, maximum urinary flow rate, and bladder compliance. Trial Sequential Analysis confirmed the superiority of EA over conventional rehabilitation. Reported adverse events were infrequent and mild. These findings suggest that EA may improve bladder function and urinary control after spinal cord injury, although further randomized trials remain necessary.

In a perspective article, Moumeni re-examined rehabilitation paradigms through an intensity-centered approach, integrating evidence from randomized clinical trials, high-repetition training, high-intensity interval training protocols, intensive military training models, and programs in resource-limited settings. Across studies, high-intensity rehabilitation performed for 4–6 h daily over 3–4 weeks produced functional improvements comparable or superior to conventional programs lasting 12–16 weeks. Neural and muscular adaptations responded more effectively to concentrated stimulation than to lower-intensity regimens. Family involvement increased therapeutic density and continuity. The findings suggest that rehabilitation outcomes depend on stimulation intensity rather than session duration, supporting efficient post-stroke recovery strategies.

Further reinforcing the growing interest in neuromodulation-based interventions for spinal cord injury rehabilitation, Benavides et al. conducted a narrative review evaluating the use of repetitive transcranial magnetic stimulation (rTMS) for motor recovery after spinal cord injury. Drawing on evidence from 14 studies, the authors found that various rTMS protocols were associated with reductions in spasticity and improvements in upper limb motor function and lower limb motor function. Despite these promising findings, substantial methodological heterogeneity and generally small sample sizes limit definitive conclusions. The review emphasizes the need for larger, well-controlled clinical trials and standardized assessment protocols to better establish the role of rTMS as a neurorehabilitation tool for individuals with spinal cord injury.

Looking toward future directions and innovative, accessible rehabilitation models, Hu et al. describe the protocol of a pilot randomized controlled trial designed to evaluate a home-based Action Observation and Motor Imagery intervention for individuals with spinal cord injury. The study aimed to assess the feasibility, acceptability, and preliminary effects of the intervention on cognitive function and depression, while also investigating underlying neural mechanisms through multimodal MRI. By targeting cognitive and emotional outcomes in addition to physical rehabilitation, this innovative approach may broaden the scope of neurorehabilitation strategies for people living with spinal cord injury.

Collectively, the studies included in this Research Topic illustrate the breadth and dynamism of contemporary neurorehabilitation and regenerative research. From advances in prognostic assessment and critical care management to emerging neuromodulatory, robotic, and regenerative interventions, the contributions presented herein highlight the importance of integrating multidisciplinary perspectives to address the complex challenges posed by traumatic brain and spinal cord injuries. Together, these findings not only advance current scientific understanding but also provide a foundation for future investigations aimed at developing more effective, personalized, and accessible rehabilitation strategies. As the field continues to evolve, the translation of innovative discoveries into clinical practice will remain essential for improving functional outcomes and quality of life for individuals affected by these devastating neurological conditions.

## References

[B1] Areas F. Z. Schwarzbold M. L. Diaz A. P. Rodrigues I. K. Sousa D. S. Ferreira C. L. . (2019). Predictors of hospital mortality and the related burden of disease in severe traumatic brain injury: a prospective multicentric study in Brazil. Front. Neurol. 10:432. doi: 10.3389/fneur.2019.00432 31105642 PMC6494964

[B2] Arêas F. Z. S. Da Silva Filho W. G. Arêas G. P. T. Jo H. J. (2024). Editorial: neurorehabilitation in neurotrauma: treating traumatic brain and spinal cord injuries. Front. Hum. Neurosci. 18:1484962. doi: 10.3389/fnhum.2024.1484962 39318701 PMC11421167

[B3] Arêas F. Z. S. Gonçalves J. V. (2022). Traumatismo crânio encefálico no Brasil: uma silenciosa e devastadora epidemia. Rev. Brasil. Pesquisa Saúde 24. doi: 10.47456/rbps.v24i1.39321

[B4] Cramer S. C. (2018). Treatments to promote neural repair after stroke and traumatic injury. Nature 557(Suppl. 7705), S53–S60.29844557

[B5] Gonçalves J. V. Lirio P. H. C. Louzada C. B. de Almeida H. S. Vasconcellos H. S. Ramos L. S. . (2025). Predictors of functional recovery in the first year after severe traumatic brain injury. Braz. J. Phys. Ther. 29:101251. doi: 10.1016/j.bjpt.2025.101251 40882296 PMC12409814

[B6] Hernandez-Navarro A. Ros-Alsina A. Yurtseven M. Wright M. Kumru H. (2025). Non-invasive cerebral and spinal cord stimulation for motor and gait recovery in incomplete spinal cord injury: systematic review and meta-analysis. J. Neuroeng. Rehabil. 22:53. doi: 10.1186/s12984-025-01557-4 40050875 PMC11887137

